# Intellectual disability associated with a homozygous missense mutation in *THOC6*

**DOI:** 10.1186/1750-1172-8-62

**Published:** 2013-04-26

**Authors:** Chandree L Beaulieu, Lijia Huang, A Micheil Innes, Marie-Andree Akimenko, Erik G Puffenberger, Charles Schwartz, Paul Jerry, Carole Ober, Robert A Hegele, D Ross McLeod, Jeremy Schwartzentruber, Jacek Majewski, Dennis E Bulman, Jillian S Parboosingh, Kym M Boycott

**Affiliations:** 1 Children’s Hospital of Eastern Ontario Research Institute, University of Ottawa, 401 Smyth Road, Ottawa, ON, K1H 8L1, Canada; 2 Department of Medical Genetics, University of Calgary, Calgary, AB, Canada; 3 Alberta Children’s Hospital Research Institute for Child and Maternal Health, Calgary, AB, Canada; 4 Department of Biology, University of Ottawa, Ottawa, ON, Canada; 5 Clinic for Special Children, Strasburg, PA, USA; 6 Greenwood Genetic Center, Greenwood, SC, USA; 7 Paul Jerry Consulting Psychology, Inc., Medicine Hat, AB, Canada; 8 Department of Human Genetics, University of Chicago, Chicago, IL, USA; 9 Robarts Research Institute and University of Western Ontario, London, ON, Canada; 10 McGill University and Genome Quebec Innovation Centre, Montreal, QC, Canada

**Keywords:** Intellectual disability, THOC6, THO/TREX complex, mRNA export, Hutterite

## Abstract

**Background:**

We recently described a novel autosomal recessive neurodevelopmental disorder with intellectual disability in four patients from two related Hutterite families. Identity-by-descent mapping localized the gene to a 5.1 Mb region at chromosome 16p13.3 containing more than 170 known or predicted genes. The objective of this study was to identify the causative gene for this rare disorder.

**Methods and results:**

Candidate gene sequencing followed by exome sequencing identified a homozygous missense mutation p.Gly46Arg, in *THOC6*. No other potentially causative coding variants were present within the critical region on chromosome 16. THOC6 is a member of the THO/TREX complex which is involved in coordinating mRNA processing with mRNA export from the nucleus. *In situ* hybridization showed that *thoc6* is highly expressed in the midbrain and eyes. Cellular localization studies demonstrated that wild-type THOC6 is present within the nucleus as is the case for other THO complex proteins. However, mutant THOC6 was predominantly localized to the cytoplasm, suggesting that the mutant protein is unable to carry out its normal function. siRNA knockdown of *THOC6* revealed increased apoptosis in cultured cells.

**Conclusion:**

Our findings associate a missense mutation in *THOC6* with intellectual disability, suggesting the THO/TREX complex plays an important role in neurodevelopment.

## Background

Intellectual disability (ID) is the most frequent handicap affecting children and it is one of the greatest challenges in healthcare as ID is associated with life-long impairments that have a profound impact on individuals, families, and society. Genetic causes of ID are diverse and include chromosomal aberrations and autosomal dominant, X-linked, autosomal recessive, and mitochondrial DNA mutations. Nonsyndromic ID is characterized by ID as the sole clinical feature in patients, while syndromic ID occurs in combination with one or more additional clinical features. Recently, next-generation sequencing of 136 consanguineous families identified 23 previously implicated ID genes and 50 novel candidate genes, confirming the suspected significant genetic heterogeneity underlying ID [1].

The Hutterites are a German-speaking Anabaptist group that arose during the Protestant Reformation (1528) in South Tyrol (Austria) [2]. The Hutterite population has been living on the North American prairies since the late 1800s, now numbers over 40,000, and is comprised of three essentially endogamous groups, Schmiedeleut, Dariusleut, and Lehrerleut. Their genetic isolation, small founder population, excellent genealogical records, large completed family size, and uptake of modern health care facilitates genetic studies [2]. Over 30 autosomal recessive conditions have been identified within this population and additional novel Mendelian disorders continue to be recognized [3].

We recently described a novel autosomal recessive ID disorder in two sets of sisters from related Dariusleut Hutterite families [4]. As previously described, their clinical features include significant learning disabilities and head circumference at the 2nd centile without apparent structural CNS malformations on MRI. All four patients share recognizable facial features including a tall forehead with high anterior hairline, deeply-set eyes with short, upslanting palpebral fissures, long nose with low-hanging columella, and thick vermilion of the upper and lower lip. Other clinical features include dental malocclusion and caries, myopia, malformations of the heart (one patient with patent ductus arteriosus and ventricular septal defect, another with ventricular septal defect only), and renal abnormalities (one patient with horseshoe kidney, another patient with left renal agenesis and renal failure identified at age 13 requiring dialysis followed by transplant at age 15 years; at diagnosis the unilateral right kidney was echogenic and atrophic on ultrasound imaging) [4]. The patients underwent neuropsychological testing to further characterize their learning disabilities and the results were consistent with moderate ID without a significant difference between verbal comprehension and perceptual reasoning. Based on the clinical presentation, this disorder is best classified as a syndromic form of ID, but due to the subtle and variable nature of the additional features the diagnosis of syndromic ID could be easily missed. Furthermore, the distinction between syndromic and nonsyndromic is becoming blurred with a subset of ID genes causing both forms of disability [5,6].

A single region on 16p13.3 was identified by genome-wide homozygosity mapping with DNA samples from the four patients using a 50K Affymetrix GeneChip SNP array followed by refinement with microsatellite markers using all available family members [4]. The maximum size of the region was 5.1 Mb with the distal boundary at 1,404,019 and the proximal boundary at 6,458,669 (NCBI Build 36.3) [4]. Sanger followed by exome sequencing identified a single potential disease-causing missense mutation in *THOC6* (*fSAP35; WDR58*). THOC6 is a part of the THO complex which is involved in coordinating mRNA processing with export. We demonstrate that the p.Gly46Arg substitution results in protein mislocalization to the cytoplasm suggesting that the mutant protein is unable to carry out an export function. Moreover, depletion of THOC6 induces apoptosis in mammalian cells. These findings indicate that the *THOC6* missense mutation perturbs protein function, supporting a disease-association between THOC6 and intellectual disability and an important role for the THO/TREX complex in neurodevelopment.

## Methods

### Patient recruitment

Institutional Research Ethics Board approval for the study reported here was obtained from the University of Calgary and the Children’s Hospital of Eastern Ontario and informed consent was obtained from responsible persons (parents) on behalf of all study participants. Total genomic DNA was extracted from blood following standard procedures.

### Sanger sequencing

Ninety-seven out of the 173 genes within the 5.1 Mb critical region on 16p13.3 (NCBI build 36.3, including hypothetical genes and pseudogenes) were PCR amplified and Sanger sequenced. These genes were chosen based on expression or function indicating a potential role in neurodevelopment; genes associated with dissimilar disorders or unrelated functions were excluded from sequencing. Primers were designed to assess the coding regions and intron-exon boundaries of the prioritized genes using the program Oligo (Molecular Biology Insights, Inc., Cascade, CO). PCR and bidirectional sequencing was performed on DNA samples from a patient, a parent, and unaffected control. Primer sequences and reaction conditions are available upon request. Sequence subtraction analysis was performed using Mutation Surveyor (SoftGenetics LLC, State College, PA).

### Exome sequencing

Exome capture and high-throughput sequencing of DNA from one patient was performed at the McGill University and Genome Québec Innovation Centre (Montréal, Canada). Exome target enrichment was performed using the Agilent SureSelect 50 Mb All Exon Kit (V3), and sequencing (Illumina HiSeq) generated 14 Gbp of 100 bp paired-end reads. An in-house annotation pipeline was used to call and annotate coding and splice-site variants. Reads were aligned to hg19 using BWA [7] and duplicate reads were marked using Picard [8] and excluded. Single nucleotide variants and short insertions and deletions (indels) were called using SAMtools mpileup [9] and bcftools and quality-filtered to require a minimum 20% of reads supporting the variant call. Variants were annotated using Annovar [10] as well as custom scripts to select coding and splice-site variants, and to exclude common (≥1% minor allele frequency) polymorphisms represented in the NHLBI exome server [11], or in 435 control exomes sequenced at the McGill University and Genome Québec Innovation Centre. Variants within the mapped region at 16p13.3 were considered as candidates. Coverage of genes within the region was assessed to determine the fraction of bases in each exon with at least 5 reads covering the position. Although average coverage was much higher, 5× was deemed sufficient to call variants within this homozygous region. To determine the proportion of CCDS exons with sufficient coverage, exons of all isoforms within the region were counted (exons present in multiple isoforms were counted only once) and exons with less than 99% coverage at 5× were deemed incomplete.

### Taqman genotyping

500 Schemiedeleut controls, 92 Daruisleut controls, and 120 Lehrerleut controls were genotyped by a TaqMan SNP genotyping assay for the variant. Life Technologies’ TaqMan genotyping protocol and mix were used following standard procedures (Forward Primer: AGAAACTTCCCACATGGTGAGAC, Reverse Primer: ATTAGCCCTGGCACTTGGC, Wild-type Probe: VIC-TG GCAACAATTACGGGC-mgb (minor groove binder), Mutant Probe: FAM-ACAATTACAGG CAGATT-mgb).

### Plasmids and site-directed mutagenesis

Wild-type (WT) *THOC6* cDNA was purchased from the Centre for Applied Genomics (Toronto). The primer sets: 5’ TTA GGA TCC ATG GAC TAC AAG GAT GAC GAT GAC AAG GAG CGA GCT GTG CCG CTC 3’/5’ GTA GCG GCC GC TCA GAA GGA CAG GGA GAA GGC TCG 3’ and 5’ TTA GGA TCC ATG GAG CGA GCT GTG CCG CTC 3’/5’ GGA GCG GCC GC TCA CTT GTC ATC GTC ATC CTT GTA GTC GAA GGA CAG GGA GAA GGC TCG 3’ were used to PCR amplify the full length *THOC6* cDNA in order to introduce a FLAG tag at the N- and C-terminus, respectively and subsequently the cDNA was subcloned into pcDNA3. Site-directed mutagenesis of *THOC6* was performed using the QuikChange II mutagenesis kit (Agilent). The mutated cDNA constructs were sequenced to confirm the fidelity of the mutagenesis reactions.

### Immunostaining

HeLa cells were seeded at 2 × 10^5^ cells/well on glass coverslips in six-well plates and transiently transfected with FLAG-tagged WT *THOC6* or mutant *THOC6* plasmids with lipofectamine 2000 (Invitrogen). Twenty-four hours after the transfection, the cells were fixed in 2% paraformaldehyde and permeabilized with PBS containing TritonX-100 (0.05%). Cells were incubated with anti-FLAG antibody (Sigma) for 1 h followed by incubation with Alexa 488-conjugated goat anti-mouse IgG secondary antibody (Invitrogen). The cells were stained with DAPI and mounted onto microscope slides. Images were obtained with a Nikon Eclipse TE2000-E system controlled and processed by EZ-C1 3.50 (Nikon) software. Two replicates and a chi-square test were performed on each replicate.

### siRNA transfection

Human *THOC6*-specific and non-targeting control siRNAs were purchased from Dharmacon. HeLa cells were plated at a density of 2 × 10^5^ cells/well on a coverslip in a six-well plate, 24 h prior to transfections. Cells were transfected with 100 nM of *THOC6*-specific or non-targeting scramble siRNA with Lipofectamine RNAiMAX (Invitrogen) and cultured for 48 h.

### TUNEL staining

TUNEL staining was performed using the *in situ* cell death detection kit, TMR red (Roche), following the manufacturer’s protocol. Two replicates and a chi-square test were performed on each replicate.

### Zebrafish whole-mount *in situ* hybridization

Whole-mount *in situ* hybridization was performed on embryos at 24, 48, and 72 h post fertilization (hpf) as described previously [12]. Briefly, full-length cDNA of zebrafish *thoc6* was obtained from Open Biosystems (EDR5649-100965848). The cDNA was cloned into pGEM-T vector (Promega) and used as a template for *in vitro* synthesis of an antisense mRNA probe. Embryos were fixed in 4% paraformaldehyde in PBS, hybridized with DIG-labeled riboprobes at 65°C, and followed by incubation with anti-DIG antibody conjugated with alkaline phosphatase (AP) (Roche). NBT and BICP were used as the substrates of AP to generate the purple coloration.

## Results and discussion

### Identification of *THOC6* as candidate gene for ID

**Figure 1:**
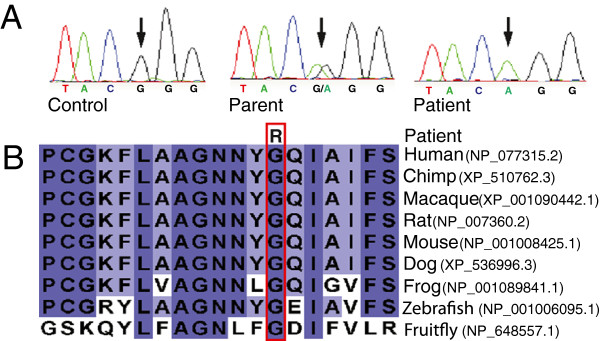
**Mutation of *THOC6*, identified in the patients with intellectual disability. A.** Sanger sequencing indicating the mutation c.136G>A (RefSeq NM_024339.3) as homozygous in the patient and heterozygous in the parent. **B.** Conservation of the glycine residue (red boxed) at the site of the glycine to arginine substitution in the patients.

Prior to the availability of whole-exome sequencing, the coding regions of 97 out of the 173 genes within the mapped region (NCBI build 36.3, including hypothetical genes and pseudogenes) were Sanger sequenced in a patient and parent. These genes were chosen based on expression or function indicating a potential role in neurodevelopment; genes associated with dissimilar disorders or unrelated functions were excluded from sequencing. A potential disease-causing missense mutation was identified in *THOC6* (fSAP35), c.136G>A (p.Gly46Arg) (RefSeq NM_024339.3) (Figure 1A). Sanger sequencing confirmed the presence of this potential mutation in the homozygous state in the other three patients.

Exome sequencing was performed to determine if any additional potentially pathogenic variants were present in the coding sequence of genes within the critical region. The mean exome read depth for the sample was 143×. Exome sequencing coverage was determined for all CCDS annotated genes (hg19) within the region and 92% of exons from all isoforms were covered completely at greater than 5×. When Sanger sequencing results were included this increased to 96% of exons being covered. Gene coverage statistics are included in Additional file 1: Table S1. Variants identified by exome sequencing were filtered to exclude common (≥1% minor allele frequency) polymorphisms represented in the NHLBI exome server [11], dbSNP, or in 435 control exomes sequenced at our center. The only rare variant in the mapped region was the *THOC6* homozygous variant c.136G>A, which was not present in any of the controls.

The c.136G>A variant was found in the heterozygous state in the parents of the patients and none of the six unaffected siblings available for testing were homozygous. The variant was not seen in 150 controls from the general population. Frequency of the variant was determined in the three Hutterite leuts by Taqman genotyping. The variant was seen at a frequency of 3% in 92 Dariusleut controls and at a frequency of 2% in 120 Lehrerleut controls; no homozygous controls were identified. The variant was not seen in 500 Schmiedeleut controls indicating it is likely shared only between the Dariusleut and Lehrerleut and that additional affected individuals may exist within these communities. The glycine at position 46 is highly conserved between species and occurs within a relatively conserved region (Figure 1B), suggesting that this amino acid plays an important role in THOC6. Polyphen [13] and SIFT [14] predicted *THOC6* p.Gly46Arg to be damaging.

Next, we sequenced *THOC6* in a collection of 140 female patients with ID and microcephaly; a female patient cohort was used to enrich for non X-linked ID. No rare homozygous or compound heterozygous variants considered likely to be disease-causing were identified.

THOC6 is a part of the THO complex which is involved in coordinating mRNA processing with export. In humans, this complex is comprised of THOC1, THOC2, THOC5, THOC6, and THOC7 [15]. The THO complex components also interact with UAP56, ALY, and TEX forming the larger TREX complex (transcription export complex) [15-17]. In yeast, this complex was found to have a role in coupling transcription and polyadenylation to mRNA export with mutants showing defects in transcription, polyadenylation, nuclear accumulation of poly (A) RNA, the formation of heavy chromatin, and accumulation of stalled nuclear pore components [17-19]. The necessity of the THO complex for export of individual transcripts in yeast has been linked to genes with strong promoters and rapid transcription rates [20,21]. Initial studies in human cell lines found THOC1 to bind DNA and interact with RNA Polymerase II as in yeast [22]; however, recent literature has favoured the hypothesis that in metazoan organisms the THO complex couples mRNA processing with export. In metazoans, the THO complex components have only been found bound to processed transcripts [15] and export of transcripts to the cytoplasm by THO was determined to be both cap and splicing dependent [23].

### Impaired cellular localization of mutant THOC6 p.Gly46Arg

**Figure 2:**
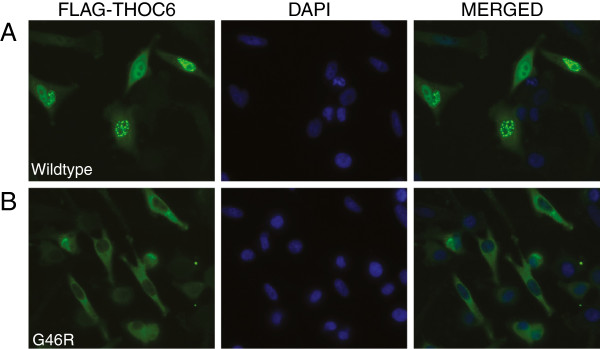
**Cellular localization of transfected WT and p.Gly46Arg THOC6.** Left panels, HeLa cells were transiently transfected with FLAG-tagged WT THOC6 **(A)** or THOC6 p. G46R **(B)**. Green fluorescence (Alexa Fluor 488) indicates the position of the FLAG-tagged THOC6 proteins within the cell. Middle panels, DAPI staining indicates the position of the nuclei. Right panels, merged DAPI and THOC6 images shows nuclear localization of WT THOC6 protein **(A)** whereas, the mutant THOC6 p.G46R protein was confined to the cytoplasm **(B).**

Members of the THO complex including THOC1, THOC2, THOC5, and THOC7 have been found to be localized to the nucleus and to shuttle between the nucleus and the cytoplasm [24-26]. Within the nucleus, they have been found to co-localize with splicing factors in nuclear speckle domains and function in the release of mRNA from these domains [15,27]. We subcloned the WT and mutant *THOC6* (c.136G>A) cDNA with a FLAG epitope at the C-terminus into pcDNA3 vector. Immunostaining was performed with anti-FLAG antibody on HeLa cells transfected with the WT and mutant constructs. The majority of cells showed a speckled nuclear localization of WT THOC6 protein similar to what has been seen for THOC1 and THOC2 [15,22,28] (Figure 2A), whereas the mutant THOC6 p.Gly46Arg protein was confined to the cytoplasm (Figure 2B). Similar results were obtained using N-terminal FLAG tagged proteins. The difference in localization was significant for both the C-terminal and N-terminal FLAG tagged protein (chi-square test) (Additional file 2: Figure S1). The absence of localization of the p.Gly46Arg mutant protein in the nucleus suggests that the mutant protein’s normal function may be perturbed. In HeLa cells, knockdown of *THOC2*, *THOC1*, and *THOC7* led to polyA RNA nuclear accumulation, whereas knockdown of *THOC5*, *THOC6*, and *TEX* did not [29]. Knockdown of the THO complex in *Drosophila* appears to affect the export of only a very small subset of mRNA transcripts including the rapidly transcribed inducible *HSP70* transcripts [16]. Knockdown of *THOC5* and *THOC6* was seen to lead to the retention of *HSP70* mRNA in the nucleus after heat shock [24]. Further elucidation of the role of THOC6 in mRNA export will provide important insights into the pathophysiology of this disorder.

### Depletion of THOC6 increases apoptosis in mammalian cells

**Figure 3:**
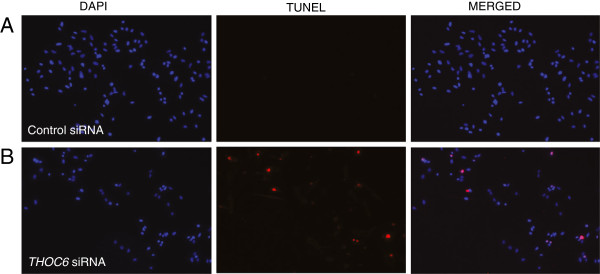
**siRNA knockdown of *THOC6 *leads to increased apoptosis.** HeLa cells transfected with *THOC6*-specific siRNA or no-targeting control siRNA were TUNEL stained. DAPI staining indicates the position of the nuclei. Knocking down of *THOC6* resulted in an increased proportion of cells undergoing apoptosis with positive TUNEL staining **(A)**, compared with the control siRNA transfected cells **(B)**.

Apoptosis occurs during brain development and is highly regulated; animal models and human disorders suggest that increased levels of apoptosis can lead to severe neurological defects including microcephaly [30-32]. There has been evidence suggesting a role of the THO complex in the survival of rapidly proliferating cells by preventing apoptosis [33,34]. We sought to determine whether loss of *THOC6* expression causes apoptosis in a mammalian cell line. *THOC6* gene-specific siRNAs were used to knockdown *THOC6* in HeLa cells. A robust decrease in the levels of THOC6 protein in HeLa cells transfected with *THOC6* gene-specific siRNAs (Additional file 3: Figure S2A) was observed. TUNEL staining was performed to examine the level of apoptosis. A significantly increased proportion of cells with positive TUNEL staining was seen in cells transfected with *THOC6* siRNAs, compared to the cells transfected with a control siRNA (Figure 3 and Additional file 3: Figure S2B), indicating that loss of THOC6 leads to an increase in apoptosis.

### Expression of *Thoc6* in the central nervous system during zebrafish embryonic development

**Figure 4:**
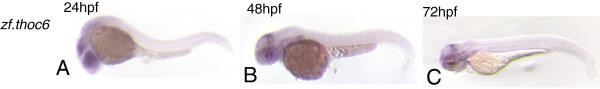
**Expression of zebrafish *thoc6 *in the brain.** Expression of zebrafish *thoc6* was assessed by whole mount *in situ* hybridization. Side views of whole mount *thoc6* expression observed in 24, 48 and 72 hpf WT embryos. *thoc6* is highly expressed in the developing optic tectum and the eyes at 24 hpf **(A)**. This expression decreases at 48 **(B)** and 72 hpf **(C)** but a low level of expression is observed at the midbrain-hindbrain boundary.

Genes implicated in neurodevelopment and ID are diverse in function and many are not limited to central nervous system expression, but detailed expression studies of *THOC6* had not yet been performed. We examined expression of the zebrafish *thoc6* ortholog during embryonic development. Zebrafish Thoc6 shares 59% protein sequence identity with human THOC6, and the p.Gly46 in humans is conserved in the zebrafish sequence. *In situ* hybridization revealed that *thoc6* mRNA is highly expressed in the developing midbrain and the eyes at 24 h post fertilization (hpf) and the expression becomes restricted afterwards to the posterior part of the midbrain and the midbrain-hindbrain boundary (Figure 4). This expression pattern implicates an important role for *THOC6* in neurodevelopment, which is of interest considering the central clinical manifestation in the patients is intellectual disability. Given the other, more variable, clinical manifestations observed in the patients, it is likely that THOC6 also has a role in the development of other systems, particularly the heart and kidney.

Well-characterized causes of non-syndromic ID include genes encoding for synaptic proteins, neuronal specific proteins, and those involved in neurotransmitter release [5]. Other causes of ID are not neural specific genes, but genes coding for proteins that participate in defined processes known to be important for brain function such as metabolism and cell adhesion [5,35-37]. Other identified pathways include the Rho GTPases that play a role in regulating the actin cytoskeleton [38], the ERK/MAPK pathway that responds to growth factors [5], and the NF-κB transcription regulation pathway [39-41]. Genes causing microcephaly with ID include centrosomal, cell cycle, and DNA damage repair genes [42,43]. Other ID genes appear to function in fundamental cellular processes, yet give rise to disorders where the predominant or only feature is ID [1,44]; this includes genes involved in mRNA processing such as *ZC3H14*[45]. *THOC6*, as part of a pathway involved in mRNA export and protection against apoptosis, is best classified with this latter group of genes implicated in fundamental cellular processes, though in keeping with the predominant feature of this syndrome, the highest level of expression of *THOC6* is in the developing brain.

## Conclusion

In the current study, we have shown that a mutation resulting in THOC6 loss-of-function is associated with a syndromic form of autosomal recessive ID in the Hutterite population. The p.Gly46Arg substitution results in protein mislocalization to the cytoplasm. Moreover, depletion of THOC6 induces apoptosis in mammalian cells. In zebrafish, *thoc6* mRNA is highly expressed in the developing central nervous system during embryonic development. Collectively, these findings indicate that *THOC6* plays an important role in human neurodevelopment. Given that THOC6 is a member of the THO complex, mutations in other complex members may explain a portion of intellectual disability.

### Web resources

NHLBI Exome Variant Server http://evs.gs.washington.edu/EVS/

Picard http://picard.sourceforge.net/

SAMtools http://samtools.sourceforge.net/

UCSC Genome Browser http://genome.ucsc.edu/cgi-bin/hgGateway/

SIFT http://sift.bii.a-star.edu.sg/

PolyPhen-2 http://genetics.bwh.harvard.edu/pph2/

## Abbreviations

ID: Intellectual disability; Mgb: Minor groove binder; THOC6: THO complex 6 homolog; TREX Complex: Transcription export complex; CCDS: Consensus CDS; SIFT: Sorting intolerant from tolerant; PolyPhen-2: Polymorphism phenotyping; WT: Wild-type.

## Competing interests

The authors declare that they have no competing interests.

## Authors’ contributions

CLB, LH, DEB, AMI, KMB, and JSP designed the study. CLB performed Sanger sequencing. JS and JM carried out the analysis of the next-generation sequencing data. RAH, CO, and EGP performed genotyping. CLB and LH performed mammalian cell and zebrafish experiments. JSP supervised Sanger sequencing, MAA supervised zebrafish experiments, DEB supervised mammalian cell experiments, and KMB coordinated all components. CS provided cohort of ID patients. AMI and DRM provided clinical examinations and PJ provided subspecialist consultation services. CLB and LH wrote the manuscript and all authors read and approved the manuscript.

## Supplementary Material

Additional file 1: Table S1 Coverage of genes within the mapped region by exome and Sanger sequencing. CCDS genes within the mapped region (including multiple gene isoforms) are listed indicating mean coverage by exome sequencing, percentage of bases covered greater than 5× by exome sequencing, and whether the gene was Sanger sequenced. Click here for file

Additional file 2: Figure S1 THOC6 p.Gly46Arg mutation leads to statistically significant localization change compared to WT in transfected cells. Cells were classified into categories based on predominant area of THOC6 localization: nuclear localization, cytoplasmic localization, and presence in both the nucleus and cytoplasm. Sample sizes were WT C-FLAG n = 216, p.G46R C-FLAG n = 206, WT N-FLAG n = 272, p.G46R N-FLAG n = 242, and the Chi-Square test indicated a significant difference (p < 0.0001). Click here for file

Additional file 3: Figure S2 siRNA knockdown of *THOC6* leads to statistically significant increase in apoptosis in HeLa cells. A. A robust decrease in the levels of THOC6 protein in HeLa cells transfected with THOC6-specific siRNAs was seen. B. Comparison of apoptosis between control siRNA and *THOC6* siRNA transfected cells. Cells that were positive for apoptosis based on TUNEL staining were counted (n = 2500). 5.7% of the *THOC6* siRNA transfected cells were TUNEL positive compared to 0.7% of cells transfected with control siRNA. The Chi-Square test indicated this to be a significant increase (p < 0.0001). Click here for file
