# Supplementary figures and images for: Intellectual disability associated with a homozygous missense mutation in THOC6
Source: Orphanet J Rare Dis. 2013 Apr 26;8:62. doi: 10.1186/1750-1172-8-62 (PMC3644499; doi:10.1186/1750-1172-8-62)

# Supplemental Figure 1

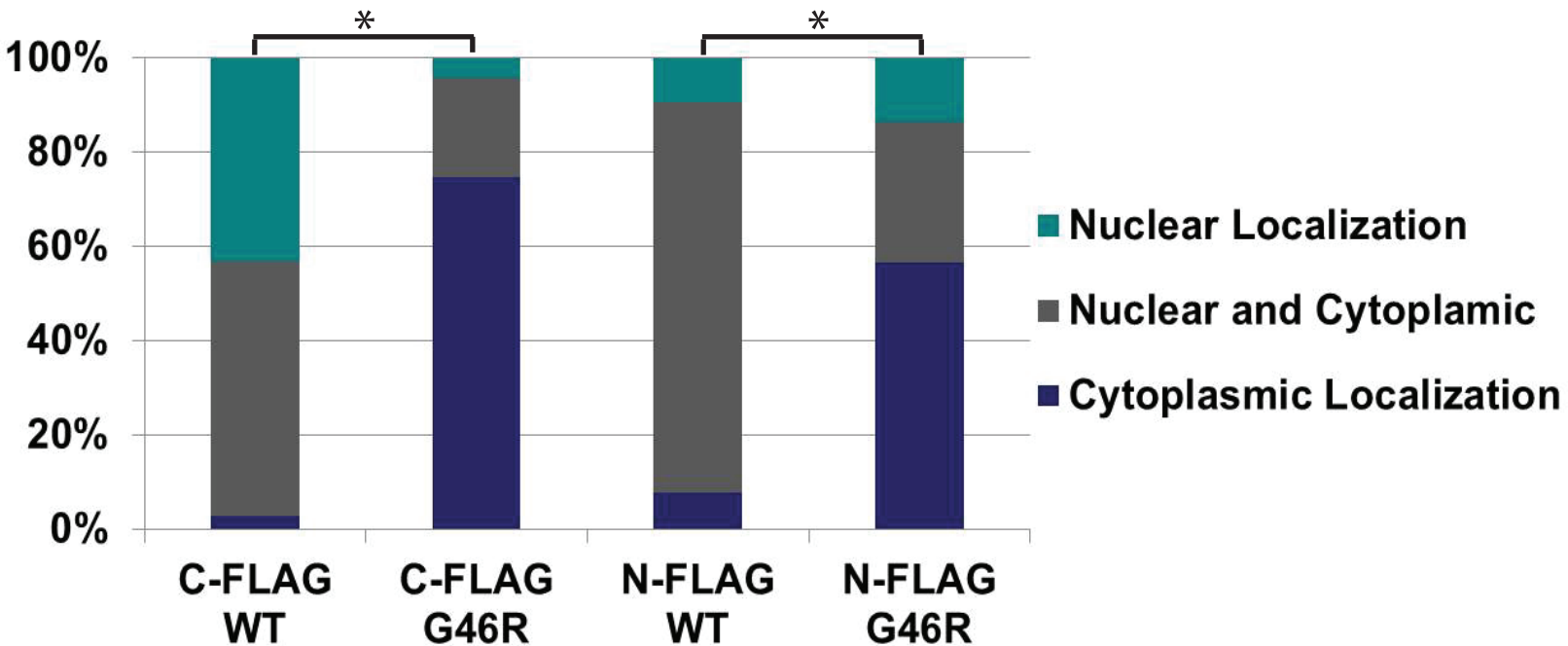

Supplement: Additional file 2: Figure S1 — THOC6 p.Gly46Arg mutation leads to statistically significant localization change compared to WT in transfected cells. Cells were classified into categories based on predominant area of THOC6 localization: nuclear localization, cytoplasmic localization, and presence in both the nucleus and cytoplasm. Sample sizes were WT C-FLAG n = 216, p.G46R C-FLAG n = 206, WT N-FLAG n = 272, p.G46R N-FLAG n = 242, and the Chi-Square test indicated a significant difference (p < 0.0001). [file 1750-1172-8-62-S2.pdf]

## Supplemental Figure 2

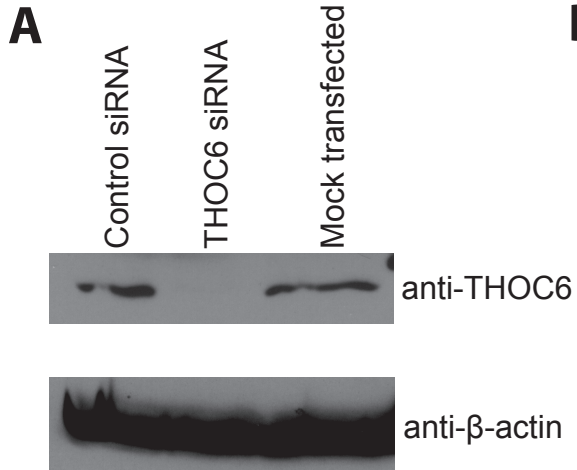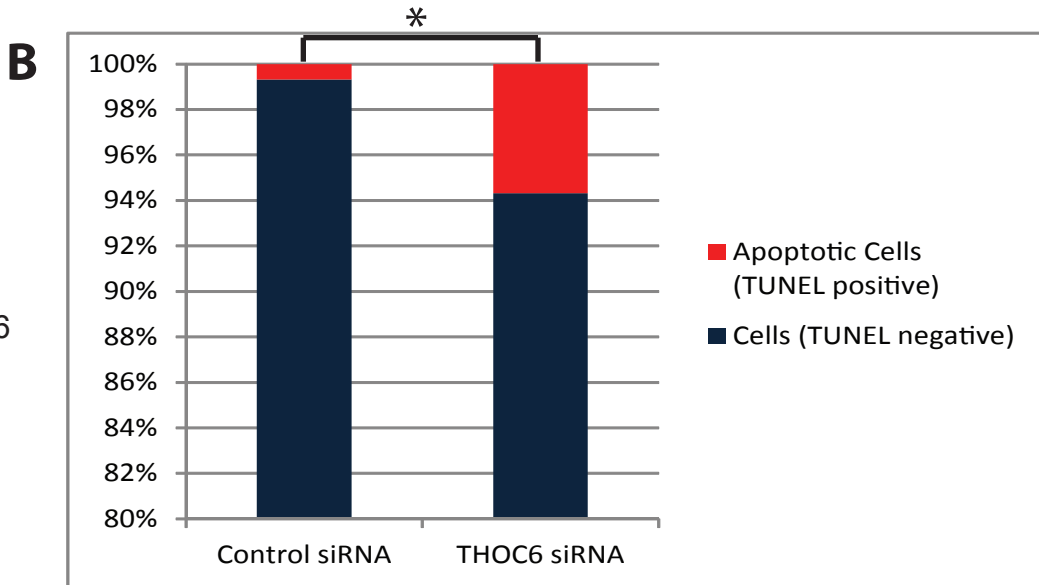

Supplement: Additional file 3: Figure S2 — siRNA knockdown of THOC6 leads to statistically significant increase in apoptosis in HeLa cells. A. A robust decrease in the levels of THOC6 protein in HeLa cells transfected with THOC6-specific siRNAs was seen. B. Comparison of apoptosis between control siRNA and THOC6 siRNA transfected cells. Cells that were positive for apoptosis based on TUNEL staining were counted (n = 2500). 5.7% of the THOC6 siRNA transfected cells were TUNEL positive compared to 0.7% of cells transfected with control siRNA. The Chi-Square test indicated this to be a significant increase (p < 0.0001). [file 1750-1172-8-62-S3.pdf]
